# Mitochondrial creatine kinase 1 in non-small cell lung cancer progression and hypoxia adaptation

**DOI:** 10.1186/s12931-021-01765-1

**Published:** 2021-07-01

**Authors:** Ming Li, Huan Liu, Juan Li, Shuai Guo, Yan Lv

**Affiliations:** 1 Thoracic Surgery, Shandong Provincial Chest Hospital, Jinan, Shandong China; 2 College of Life Sciences, Shandong Agricultural University, Tai’an, 271018 China; 3 Department of Pathology, The Third Affiliated Hospital of Shandong First Medical University and The Fourth Hospital of Jinan, Jinan, Shandong China; 4 Department of Medical Oncology, Shandong Provincial Chest Hospital, Jinan, Shandong China; 5 Department of Internal Medicine Ward IV, Shandong Provincial Chest Hospital, 12# Lieshishandong Road, Jinan, Shandong China

**Keywords:** Hypoxia, NSCLC, Mitochondrial creatine kinase 1, HIF-1, Proliferation

## Abstract

**Background:**

Hypoxia is a prominent feature of solid cancer. This research aims to expose the role of mitochondrial creatine kinase 1 (CKMT1) in non-small cell lung cancer (NSCLC) progression and hypoxia adaptation.

**Methods:**

The mRNA and protein expression of CKMT1 in NSCLC tissues were detected by using GEPIA web, immunohistochemistry and qRT-PCR. For hypoxia, cells were exposed to the 1% O_2_ atmosphere. The protein levels of HIF-1α and CKMT1 in H1650 and H1299 cells exposed to hypoxia were determined by western blot. The roles of CKMT1 on the proliferation, invasion and hypoxia adaptation of NSCLC cells were measured by CCK8, colony formation and transwell assays. Luciferase activity assay and HIF1 specific inhibitor (LW6) assay indicated the related function of hypoxia and CKMT1.

**Results:**

CKMT1 was highly expressed in NSCLC tissues, and the high level of CKMT1 was significantly correlated with the high pathological grade of NSCLC. Knockdown of CKMT1 inhibited the cell proliferation and invasion of H1650 and H1299 cells, which could be rescued by hypoxia. Hypoxia induced the accumulation of HIF-1α and the expression of CKMT1 in H1650 and H1299 cells. Furthermore, HIF-1 as a transcription factor of CKMT1, could up-regulated the expression of CKMT1 under hypoxia.

**Conclusions:**

In summary, CKMT1 has the potential as a target for NSCLC hypoxic targeted therapy.

## Background

There are more than 1.30 million newly diagnosed lung cancer cases and more than 1.20 million deaths every year worldwide [1]. Although the early diagnosis and therapeutic methods of non-small cell lung cancer (NSCLC) patients have been greatly improved, the overall 5-year survival rate has not improved significantly due to primary or secondary drug resistance [2].

Hypoxia is a prominent feature of solid cancer, mainly due to the rapid proliferation of tumor cells and the abnormal formation of blood vessels [3]. In NSCLC, hypoxia is an important factor affecting treatment resistance and poor survival [4]. Tumor cells change the expression of specific genes and activate stress response pathways to facilitate cell proliferation and growth and confer aggressive phenotype under hypoxia [5]. Hypoxia is an attractive therapeutic target, but it has not been successfully developed in most cancers including NSCLC [6]. Hypoxia inducible factor-1 (HIF-1) is a core transcriptional regulator that responds to hypoxia [7].

Mitochondrial creatine kinase 1 (CKMT1) is responsible for transferring the phosphate group of mitochondrial ATP to creatine (Cr). Recent studies have shown that CKMT1 plays different roles in a variety of tumor types. The expression of CKMT1 is significantly up-regulated in hepatocellular carcinoma [8]. CKMTI promotes the proliferation and migration of nasopharyngeal carcinoma cells, and affects the sensitivity to radiation [9]. However, it is down-regulated during the carcinogenesis of oral cancer [10] or prostate cancer [11]. However, the effect of CKMT1 on the progression of NSCLC has not been studied so far.

In this study, we will reveal the differential expression of CKMT1 in NSCLC tissues and how it participates in the regulation of the hypoxic microenvironment on the malignant progression of tumors.

## Materials

### Tumor sample collection

32 pairs of NSCLC tissues and corresponding adjacent (normal) tissues came from patients who were diagnosed with NSCLC and underwent surgical resection at Shandong Provincial Chest Hospital. All tissue samples were diagnosed by histopathology. The study was approved by the Institutional Review Committee of Shandong Chest Hospital, and the patient's written informed consent was obtained.

### RT-qPCR

Total RNA from tissues was isolated by using the TRIzol reagent (Invitrogen, Carlsbad, CA). The cDNA was synthetized with the PrimeScript Reverse Transcription Reagent Kit. RT-qPCR was performed using the SYBR Premix Ex Taq™. β-actin acted as the endogenous control. The specific primers of *CKMT1* and *β-actin* were as follows, F: 5′-CTTCACCTCACTTTACCTTC-3′, R: 5′-TCTTTTACTTCTCTGCGTCT-3′ and F: 5′-CGTGACATTAAGGAGAAGCTG-3′, R: 5′-CTAGAAGCATTTGCGGTGGAC-3′. The relative levels of CKMT1 mRNA were calculated with 2^−△△Ct^ method.

### Immunohistochemistry (IHC)

All samples were fixed in formalin and embedded in paraffin. The paraffin samples were cut into 5 μm sections. All tissues were stained using the streptavidin-peroxidase immunohistochemistry. Briefly, sections were deparaffinized in xylol and rehydrated in gradient ethanol. Subsequently, the sections were immersed in a sodium citrate solution (pH 6.0) and subjected to microwaved treatment. To eliminate nonspecific staining, the slides were incubated with 5% goat serum for 1 h. Then, the slides were incubated with rabbit polyclonal CKMT1 antibody (anti-CKMT1, 1:200, 15346–1-AP, Proteintech) for 2 h, and incubated with the labeled polymer-HRP for 1 h. DAB chromogen solution was used for color reaction, and hematoxylin was used for counterstaining.

IHC scores were quantified independently by two trained pathologists at three 200× fields. The score for each sample was multiplied by the staining intensity score and the percentage score of positive cells. The staining intensity of CKMT1 was scored as 0 (negative), 1 (weak), 2 (moderate), and 3 (strong). The percentage of positive cells was scored as 0 (0%), 1 (1–25%), 2 (26–50%), 3 (51–75%), and 4 (76–100%). A score > 3 points was considered to CKMT1 high expression.

### Cell culture, transfection and LW6 treatment

The human normal pulmonary epithelial cell line (Beas-2B) and four NSCLC cell lines (H1650, H1299, A549 and H524) were purchased from American Type Culture Collection (ATCC, Manassas, VA). All cell lines were cultured in RPMI-1640 medium (Sigma Chemical Co, St Louis, MO) supplemented with 10% fetal bovine serum (FBS, Sigma), 10 U/mL penicillin and 10 μg/mL streptomycin in a humidified atmosphere of 5% CO_2_/21% O_2_ at 37 °C. Cells were exposed to a hypoxic chamber (MACS V A500 microaerophilic workstation, Don Whitley Scientific, Bingley, UK) with atmosphere containing 5% CO_2_, 1% O_2_ and residual N_2_ at 37 °C to hypoxic conditions.

Specific siRNA sequences targeting CKMT1 (si-CKMT1) were synthetized by Genepharm Co. (Shanghai, China), and were transfected into cells using Lipofectamine 2000 reagent (Invitrogen, Carlsbad, CA). Briefly, two hours before transfection, the cell confluence reached 70% in the 35-mm dishes, and the medium was replaced with a serum-free and antibiotic-free medium. 5 μg siRNA oligomer and 5 μl Lipofectamine 2000 reagent were diluted in 250 μl serum-free and antibiotic-free culture medium, respectively. After mixing, incubate at room temperature for 5 min. The diluted siRNA oligomer and Lipofectamine 2000 reagent were mixed and incubated at room temperature for 20 min. The mixture was added to the cell culture medium and incubated at 37 °C for 6 h. Subsequently, the medium was replaced with complete medium, and subsequent processing was performed. The negative control (NC) group was transfected with meaningless siRNA sequence. The CKMT1-specific siRNA sequences were designed as follows: 5′-ACGGTACCATGGCTGGTCCCTTCTCCCGT-3′.

The cells in the 35-mm dish were treated with 10 μm LW6 and cultured under hypoxia for 24 h. The negative control (NC) group was cultured normally under normoxia.

### Western blot

Total protein was lysed using RIPA Buffer (Beyotime, Beijing, China). The equal amounts of proteins (20–40 μg/lane) were separated by SDS-PAGE and transferred to PVDF membranes (EMD Millipore, Billerica, MA). The membranes were blocked with 5% nonfat milk for 1 h and incubated with the following primary antibodies: anti-CKMT1 antibody (1:1000, 15346-1-AP), anti-HIF-1α antibody (1:1000, 20960-1-AP), anti-E-cadherin antibody (1:2000, 20874-1-AP), anti-N-cadherin antibody (1:2000, 22018-1-AP), anti-Snail1 antibody (1:2000, 13099-1-AP), and anti-Tubulin antibody (1:5000, 11224-1-AP), at 4 °C overnight. All antibodies were purchased from Proteintech. Tubulin was used as an endogenous control. Then, the membranes were incubated with an appropriately HRP-conjugated secondary antibody for 1 h and visualized using the ECL system (GE Healthcare, Little Chalfont, UK).

### CCK8 assay

The transfected cells were seeded into each well of a 96-well plate at a density of 5 × 10^3^ cells/well, and the cells were incubated for 24 h under normoxia or hypoxia. Each treatment set of 6 multiple holes. After incubating for a specified time, the old medium was removed. Fresh medium containing 10% CCK8 reagent was added to each well and incubated for 2 h. The absorbance of each well was measured at 450 nm using a microplate reader (Bio-Rad, Hercules, California, USA).

### Clone formation

The transfected cells were seeded into each well of a 6-well plate at a density of 500 cells/well, and cultured under normoxia or hypoxia for 24 h. After another 2 weeks of culture, visible colonies were formed. The colonies were fixed with methanol and stained with 0.1% crystal violet. Colonies were analyzed by Elispot system (CTL) and pictured.

### Transwell

The Matrigel-coated Transwell chamber (pore size of 8 μm) was used to evaluate cell invasion. The transfected cells were suspended in FBS-free RPMI-1640 medium and seeded into the upper chamber at a density of 2 × 10^3^ cells/well, and the lower chamber was filled with RPMI-1640 medium containing 10% FBS. The invasion cells on the lower surface of the chambers were fixed by 4% paraformaldehyde and stained with 0.1% crystal violet. Finally, the cells were counted in five randomly selected fields under an inverted microscope (200× magnification, Nikon TE2000).

### Luciferase activity assay

The CKMT1 wild type (WT) or mutant type (MUT) promoter sequence was cloned into pGL3.0 vector, and the pGL3.0 recombinant plasmid was transfected into H1650 and H1299 cells using Lipofectamine 2000. After 48 h of culture under normoxia or hypoxia, the cells were harvested and luciferase activity was detected using a luciferase assay system (Promega, Madison, Wisconsin).

### Statistical analysis

Statistical analysis was performed using GraphPad Prism Software. Each experiment was carried out 3 times independently. Data are presented as the mean ± SD. The differences between the two groups were calculated using a two-tailed Student's t-test followed by log-rank test. The comparison between the different groups were analyzed by ANOVA and followed by Fisher's exact test. P < 0.05 was considered statistically significant.

## Results

### CKMT1 is highly expressed in NSCLC tissues and cell lines

**Fig. 1:**
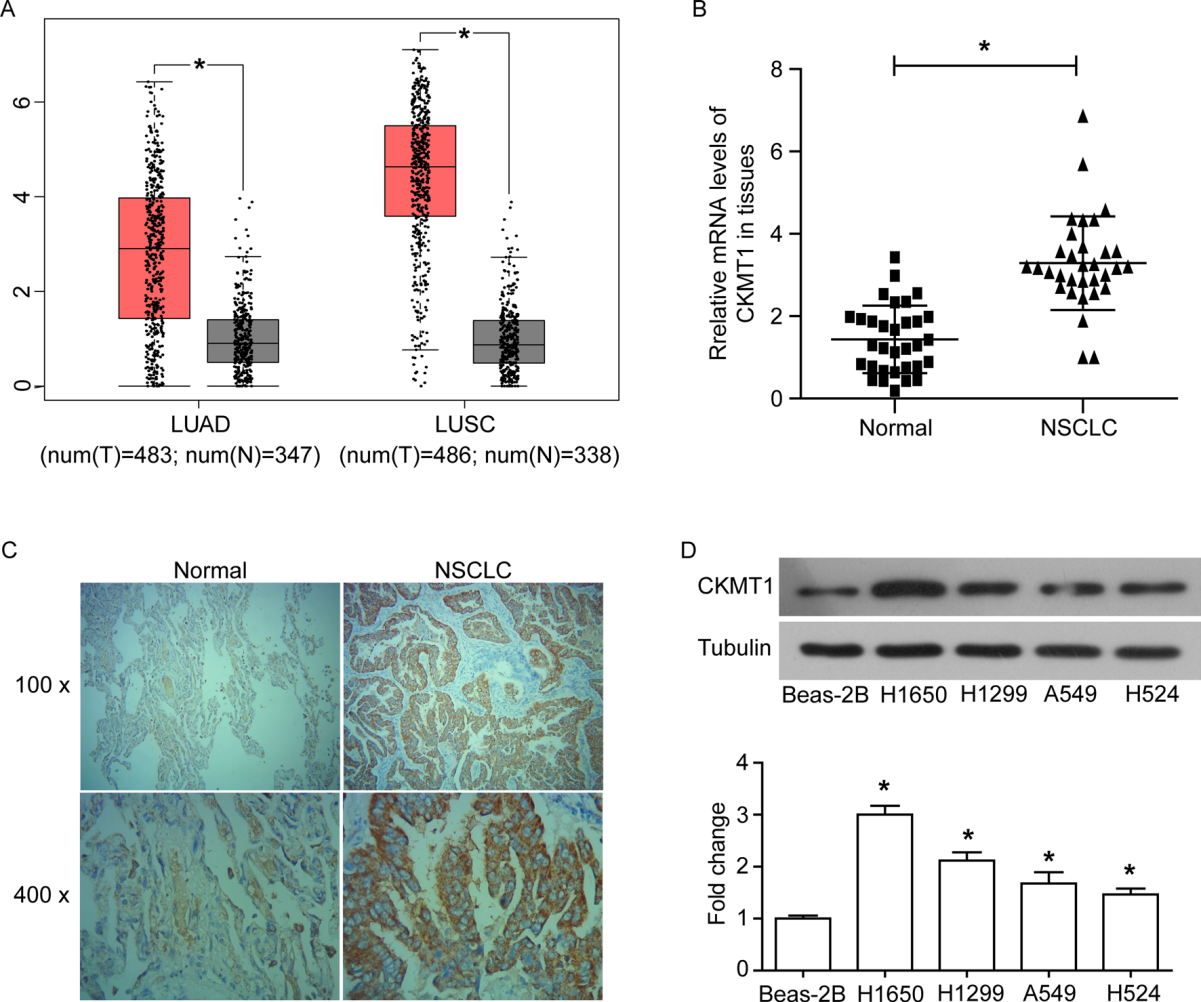
CKMT1 is highly expressed in NSCLC tissues and cell lines. **A** The boxplot of CKMT1 mRNA expression. Red boxes represent lung adenocarcinoma (LUAD) (n = 483) or lung squamous cell carcinoma (LUSC) (n = 486) tissues, while gray boxes represent normal tissues, respectively. The data came from the GEPIA database. The mRNA (**B**) and protein (**C**) expression of CKMT1 in NSCLC tissues and adjacent normal tissues was examined by qRT-PCR and immunohistochemical (IHC) analysis, respectively. (**D**) The protein expression of CKMT1 in NSCLC cell lines (H1650, H1299, A549 and H524) and normal pulmonary epithelial cell line Beas-2B was detected by western blot. *P < 0.05

The data from GEPIA [12] showed that the mRNA levels of CKMT1 were significantly increased in lung adenocarcinoma (LUAD) (n = 483) and lung squamous cell carcinoma (LUSC) tissues (n = 486), compared with normal tissues respectively (*P* < 0.05, Fig. 1A). The mRNA expression of CKMT1 in clinically collected NSCLC and adjacent tissues samples were further surveyed by qRT-PCR, and the results consistent with the GEPIA data that CKMT1 mRNA was highly expressed in NSCLC tissues (n = 32) (*P* < 0.05, Fig. 1B). The protein expression and subcellular location of CKMT1 in NSCLC tissues also detected by IHC. The percentage of CKMT1 high expression in NSCLC tissues (20/32, 62.5%) was significantly higher than in adjacent normal tissues (6/32, 18.8%) (*P* < 0.05, Fig. 1C and Table 1). Furthermore, the results of statistical analysis indicated that the high level of CKMT1 was significantly correlated with the high pathological grade of NSCLC (*P* < 0.05, Table 2). In addition, we observed that CKMT1 was highly expressed in NSCLC cell lines (H1650, H1299, A549 and H524) compared with the normal pulmonary epithelial cell line (Beas-2B cells) (*P* < 0.05, Fig. 1D).

**Table 1:** CKMT1A expression in NSCLC tissues compared with normal lung tissues

Group	n	CKMT1A expression		P
		Low (n%)	High (n%)	
NSCLC	32	12 (37.5)	20 (62.5)	0.001^**^
Normal	32	26 (81.2)	6 (18.8)	

* *P* < 0.05, ** *P* < 0.01

**Table 2:** CKMT1A expression associated with the clinicopathological parameters of NSCLC patients

Clinicopathological parameters	n	CKMT1A Low (n%)	CKMT1A High (n%)	P
Gender				
Male	20	5 (25)	15 (75)	0.131
Female	12	7 (58.3)	5 (41.7)	
Age (years)				
< 60	19	8 (42.1)	11 (57.9)	0.780
≥ 60	13	4 (30.8)	9 (69.2)	
Tumor diameter (cm)				
< 5	15	7 (46.7)	8 (53.3)	0.522
≥ 5	17	5 (29.4)	12 (70.6)	
Pathological grade				
I–II	17	10 (58.8)	7 (41.2)	0.022^*^
II–III	15	2 (13.3)	13 (86.7)	

* *P* < 0.05, ** *P* < 0.01

### Knockdown of CKMT1 inhibits the malignant proliferation, invasion and hypoxia adaptation of NSCLC cells

**Fig. 2:**
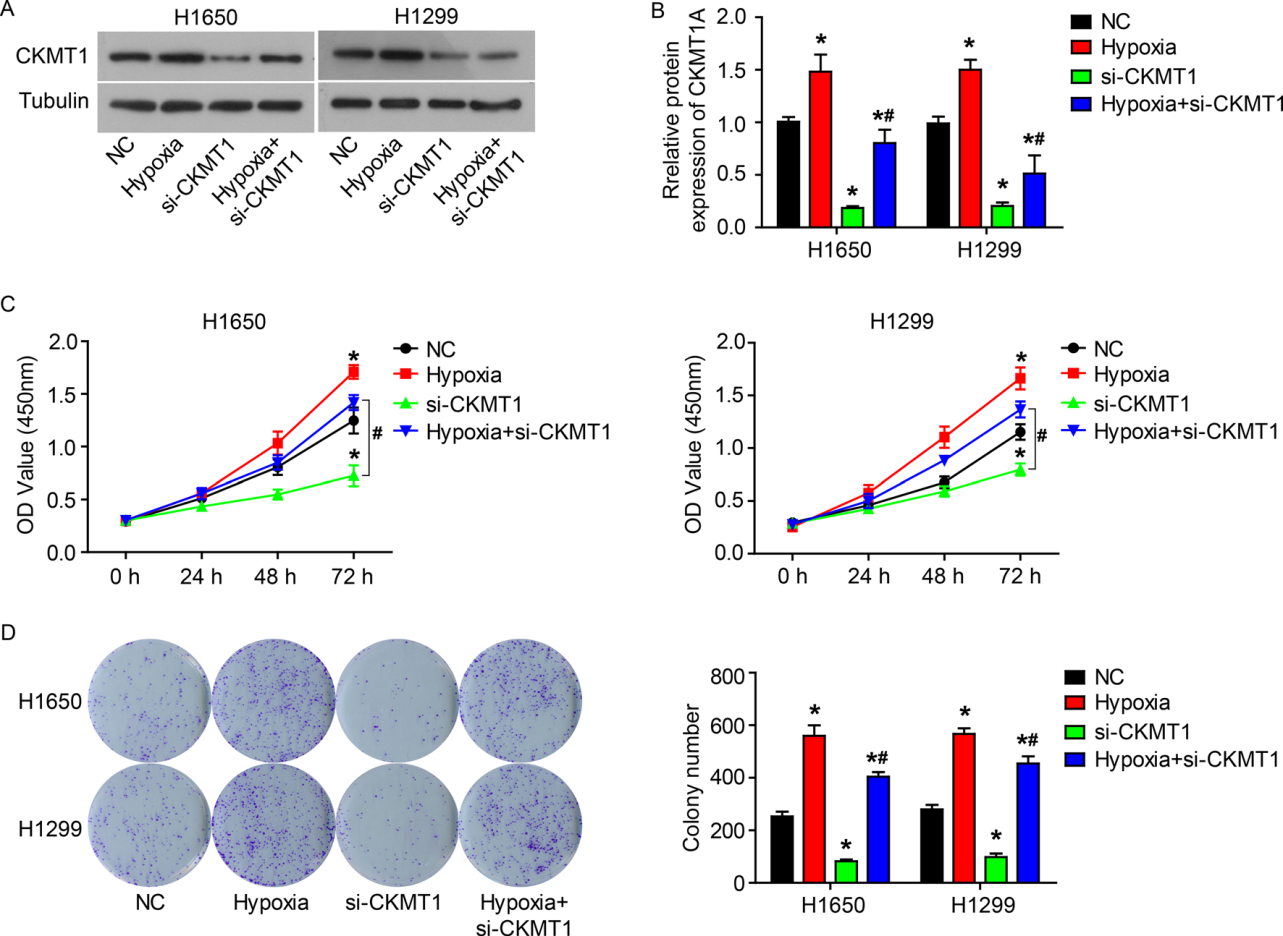
Knockdown of CKMT1 inhibits the proliferation and hypoxia adaptation of NSCLC cells. After siRNA targeting CKMT1 (si-CKMT1) was transfected into H1650 and H1299 cells and incubation for 24 h under normoxia or hypoxia, CKMT1 protein expression (**A** and **B**) by western blot, cell proliferation (**C**) and colony formation (**D**) by CCK8 and colony formation assay, respectively. *P < 0.05, compared to NC; ^#^P < 0.05, compared to si-CKMT1

**Fig. 3:**
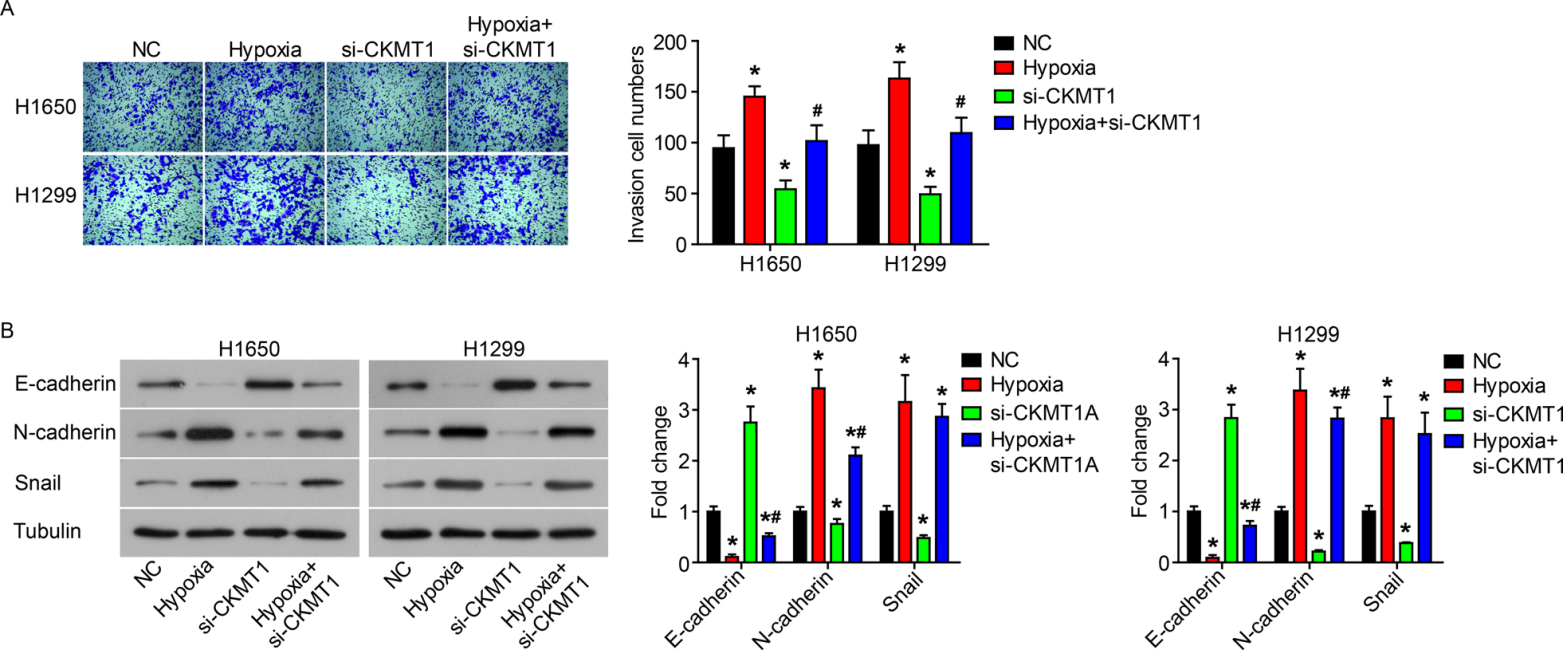
Knockdown of CKMT1 inhibits the invasion and hypoxia adaptation of NSCLC cells. After siRNA targeting CKMT1 (si-CKMT1) was transfected into H1650 and H1299 cells and incubation for 24 h under normoxia or hypoxia, the invasion ability (**A**) by Transwell assay, the expression of EMT-related proteins (**B**) by western blot. *P < 0.05, compared to NC; ^#^P < 0.05, compared to si-CKMT1

To examine the role of CKMT1 in the malignant proliferation, invasion and hypoxia adaptation of NSCLC cells, the siRNA specifically targeting CKMT1 (si-CKMT1) was used to down-regulate the expression of CKMT1 in H1650 and H1299 cells (Fig. 2A and B). Down-regulation of CKMT1 expression significantly inhibited the cell proliferation, colony formation, invasion and EMT of H1650 and H1299 cells (Figs. 2C-D and  3A–B). Furthermore, hypoxia promoted the proliferation, colony formation, invasion and EMT of H1650 and H1299 cells (Figs. 2C–D and  3A–B). In addition, we observed that the promotion of hypoxia on the malignant proliferation and invasion of H1650 and H1299 cells can be inhibited by the down-regulation of CKMT1 expression (Figs. 2C–D and  3A–B).

### Hypoxia induces the expression of CKMT1 in NSCLC cells in vitro.

**Fig. 4:**
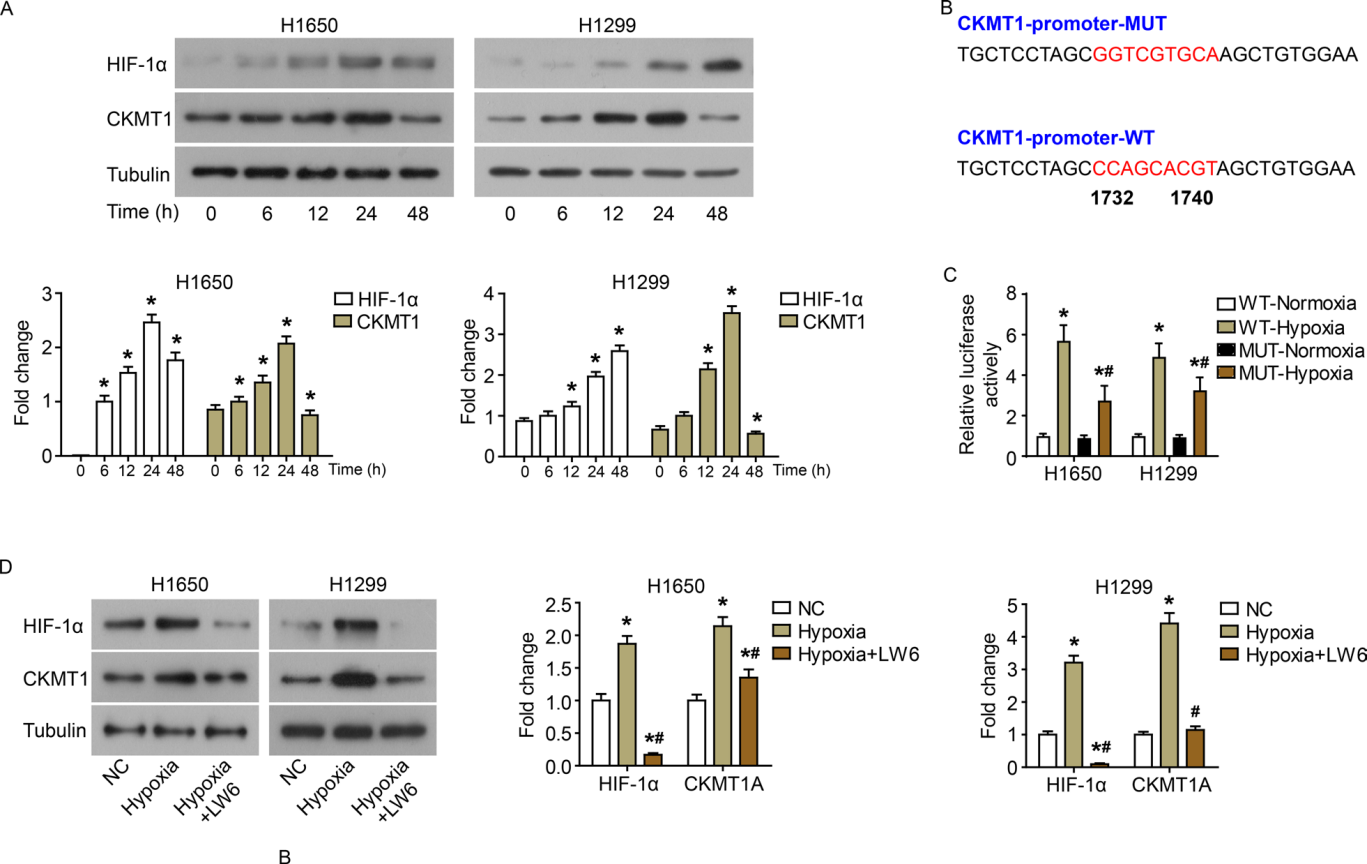
Hypoxia induces the expression of *CKMT1* in NSCLC cells. **A** The protein levels of HIF-1α and CKMT1 in H1650 and H1299 cells exposed to hypoxia for 6, 12, 24 and 48 h by western blot. **B** The wild (CKMT1-promoter-WT) or mutated (CKMT1-promoter-MUT) binding sequence of the *CKMT1* promoter region to transcription factor HIF-1 was predicted with the PROMO 3.0 website. **C** The relative luciferase activety in normoxia or hypoxia. **D** The protein levels of HIF-1α and CKMT1 in H1650 and H1299 cells exposed to hypoxia and LW6 (HIF-1α inhibitor) treatment were detected by western blot. *P < 0.05, compared to NC; ^#^P < 0.05, compared to Hypoxia

To reveal the correlation between CKMT1 expression and hypoxia adaptation, the protein levels of HIF-1α and CKMT1 were detected in H1650 and H1299 cells that exposed to hypoxia lasting for 6, 12, 24 and 48 h by western blot. As shown in Fig. 4A, hypoxia induced an increase in the protein expression of HIF-1α and CKMT1 in H1650 and H1299 cells. Until 24 h of hypoxia, the protein level of HIF-1α gradually increased with the prolongation of hypoxia; while the protein level of CKMT1 was highest at 24 h of hypoxia, and subsequently decreased. This may be due to the chronic hypoxia leading to the decline of cell viability and translation efficiency [13]. The binding sequence of the transcription factor HIF-1 to the *CKMT1* promoter region was predicted (Fig. 4B). The luciferase report assay showed that hypoxia induced the expression of wild-type exogenous CKMT1 (WT-Hypoxia) (Fig. 4C). When the HIF-1 binding site was mutated, the hypoxia-induced expression of CKMT1 (MUT-Hypoxia) was significantly reduced compared with the wild-type CKMT1 (WT-Hypoxia) (Fig. 4C). However, compared with normoxia (MUT-Normoxia), hypoxia still induced the expression of mutant CKMT1 (MUT-Hypoxia). This result means that there may be other HIF-1 binding sites, or hypoxia may regulate the expression of CKMT1 through other non-HIF-1 mechanisms. Subsequently, a specific inhibitor of HIF (LW6) was used to detect the transcriptional regulation of CKMT1 by HIF-1α. LW6 can effectively inhibited the accumulation of HIF-1α by degrading HIF-1α without affecting the level of HIF-1α mRNA under hypoxic conditions. As shown in Fig. 4D, the addition of LW6 effectively suppressed the protein levels of HIF-1α and CKMT1 in cells. In summary, as a transcription factor, HIF-1 bound to the promoter region of *CKMT1* to activate the transcription of CKMT1. Hypoxia can upregulate intracellular CKMT1 levels by inducing HIF-1α accumulation. However, hypoxia may also regulate the expression of CKMT1 through other non-HIF-1 mechanisms.

## Discussion

The cellular energy supplied by ATP closely matches the demand. Even if the load suddenly increases, the intracellular ATP level will not change drastically in the short term, which is due to the CK phosphagen system. PCr acts as a mobile energy storage reservoir for ATP regeneration. When demand continues to increase, free calcium levels rise and activate mitochondrial dehydrogenase, promoting the synthesis of nascent ATP [14]. CKMT1 is a mitochondrial creatine kinase, which plays a vital role in cells with high energy requirements or tumor cells with altered energy metabolism.

In this study, we found that the mRNA and protein expression of CKMT1 were significantly up-regulated in NSCLC tissues and cell lines. Although studies have shown that the expression of CKMT1 is significantly down-regulated in oral squamous cell carcinomas (OSCC) [10] or prostate cancer [11] tissues and cell lines. In addition, the phosphorylation of CKMT1 Y153 is generally upregulated in HER2^+^ breast cancer [15]. The differential expression of CKMT1 in different types of tumors may depend on the state of specific tumor cells, such as hypoxia, p53 mutation or methylation. For example, CKMT1 is hardly expressed in PC3 and DU145 cell lines, which is related to the lack or mutation of p53 expression, respectively [11]. In addition, the low expression of CKMT1 in OSCC-derived cell lines may be due to the frequent methylation of its CpG island region [10].

Furthermore, CKMT1 seems to have different effects on the biological function of different types of tumor cells. In present research, we found that knockdown of CKMT1 inhibited the proliferation, colony formation, invasion and EMT of H1650 and H1299 cells. In addition, inhibition of CKMT1 reduces the viability of human EVI1-positive acute myeloid leukemia (AML) cell lines, and promotes cell cycle arrest and apoptosis [16]. In addition, overexpression of CKMT1 induces the apoptosis of OSCC cells, but does not affect cell invasion [10]. This may be because the behavior of tumor cells is regulated by multiple factors and the combined effect of the tumor microenvironment. In this study, we also found that hypoxia induced the expression of CKMT1, and the induced-expression of CKMT1 was transcriptional regulated by HIF-1α accumulation. The mutation of the HIF-1 binding site in the promoter region of *CKMT1* did not completely abolish the luciferase activity. We did not find other HIF-1α binding site on the *CKMT1* promoter sequence. However, the *CKMT1* promoter sequence has other hypoxia-induced transcription factor binding sites, such as p53 and RUNX1 [16, 17]. These transcription factors may also participate in the transcriptional regulation of CKMT1 under hypoxia. Our results indicated that CKMT1 was also a target gene of HIF-1, which was reported for the first time. In addition, the transcriptional activity of HIF is determined by a variety of factors, including the presence of inhibitors and different sensitivity to HIF hydroxylation [18]. LW6 (HIF inhibitor) can effectively inhibit the accumulation of HIF-1α and the protein expression of CKMT1 in NSCLC cells.

## Conclusions

In conclusion, this study found that CKMT1 is significantly over-expressed in NSCLC. HIF-1, as a transcription factor of CKMT1, up-regulated the expression of CKMT1 under hypoxic conditions. In addition, hypoxia affects the biological function of NSCLC cells by transcriptionally regulating the expression of CKMT1.

## Data Availability

The datasets used and/or analysed during the current study are available from the corresponding author on reasonable request.

## Abbreviations

NSCLC: Non-small cell lung cancer
HIF-1: Hypoxia inducible factor-1
CKMT1: Creatine kinase 1
Cr: Creatine
IHC: Immunohistochemistry
si-CKMT1: SiRNA sequences targeting CKMT1
WT: Wild type
MUT: Mutant type
LUAD: Lung adenocarcinoma
LUSC: Lung squamous cell carcinoma
HRE: Hypoxia response element
AML: Acute myeloid leukemia
